# *Toxoplasma gondii* Infection and Suicidal Behavior in People with Alcohol Consumption

**DOI:** 10.3390/pathogens10060734

**Published:** 2021-06-10

**Authors:** Cosme Alvarado-Esquivel, Sergio Estrada-Martínez, Alma Rosa Pérez-Álamos, Isabel Beristain-García, Ángel Osvaldo Alvarado-Félix, Gustavo Alexis Alvarado-Félix, Antonio Sifuentes-Álvarez

**Affiliations:** 1 Biomedical Research Laboratory, Faculty of Medicine and Nutrition, Juárez University of Durango State, Avenida Universidad S/N, Durango 34000, Mexico; angel_alvarado_19@anglodurango.edu.mx (Á.O.A.-F.); gustavo_alvarado_19@anglodurango.edu.mx (G.A.A.-F.); dr_sifuentes@ujed.mx (A.S.-Á.); 2 Institute for Scientific Research “Dr. Roberto Rivera-Damm”, Juárez University of Durango State, Avenida Universidad S/N, Durango 34000, Mexico; sergio.estrada@ujed.mx (S.E.-M.); alma.perez@ujed.mx (A.R.P.-Á.); 3 Facultad de Enfermería y Obstetricia, Juárez University of Durango State, Blvd. Juan Pablo II 512, Durango 34000, Mexico; isabel.beristain@ujed.mx

**Keywords:** suicidal behavior, cross-sectional study, alcohol consumption, seroprevalence, epidemiology

## Abstract

We determined the association between *T. gondii* infection and suicidal behavior in people with alcohol consumption. One-thousand four-hundred and twenty-three people with alcohol consumption were screened for suicidal behavior and tested for anti-*T. gondii* IgG and IgM antibodies using commercially available enzyme-linked immunosorbent assays. Anti-*T. gondii* IgG antibodies were found in 34 of 224 (15.2%) individuals with suicidal ideation and in 118 (9.8%) of 1199 individuals without suicidal ideation (OR: 1.63; 95% CI: 1.08–2.47; *p* = 0.01). Seropositivity to *T. gondii* was associated with suicidal ideation in women (OR: 2.24; 95% CI: 1.33–3.78; *p* = 0.001) and in individuals aged ≤30 years (OR: 2.68; 95% CI: 1.22–5.87; *p* = 0.01) and >50 years (OR: 2.85; 95% CI: 1.19–6.77; *p* = 0.01). Anti-*T. gondii* IgG antibodies were found in 17 of 136 (12.5%) individuals with suicide attempts and in 135 (10.5%) of 1287 individuals without suicide attempts (OR: 1.21; 95% CI: 0.71–2.08; *p* = 0.47). Seroprevalence of *T. gondii* infection was associated with suicide attempts in women (OR: 1.88; 95% CI: 0.99–3.55; *p* = 0.04). No association between anti-*T. gondii* IgM and suicidal ideation or suicide attempts was found. Results suggest that *T. gondii* infection is associated with suicidal behavior in people with alcohol consumption.

## 1. Introduction

*Toxoplasma gondii* (*T. gondii*) is an apicomplexan protozoan [1]. Infections with *T. gondii* are common in humans and animals worldwide [2]. About 30% of the world’s population is infected with *T. gondii* [3]. Toxoplasmosis, the disease caused by *T. gondii*, is considered a neglected parasitic infection requiring public health action [4]. Infection with *T. gondii* is mainly acquired by ingestion of food or water that is contaminated with oocysts shed by cats or by eating undercooked or raw meat containing tissue cysts [5]. Clinically, infection with *T. gondii* is usually asymptomatic in immunocompetent individuals; however, in immunocompromised patients or an infected fetus, it may cause devasting effects [6]. Lymphadenopathy, ocular disease and encephalitis are some of the clinical features of toxoplasmosis [5]. However, *T. gondii* has been linked to behavioral changes in humans [7]. Infection with *T. gondii* has been associated with schizophrenia [8,9], mixed anxiety and depressive disorder [10], anxiety, obsessive–compulsive disorder, autism [11], and greater impulsivity and aggressiveness [12]. Several studies have linked *T. gondii* infection and self-harm and suicidal behaviors [13,14,15,16,17,18]. Suicide is an important public health concern that takes around 800,000 lives globally every year [19]. Alcohol consumption is a well-recognized factor associated with suicidal behavior [20,21,22,23]. Ingestion of alcohol may occur prior to suicide, as shown in a recent study where alcohol in blood was detected in 30.2% of suicide victims [24]. There is currently no information about the link between *T. gondii* infection and suicidal behavior in persons with alcohol consumption. Therefore, we sought to determine the association between *T. gondii* exposure and suicidal behavior in a sample of people with alcohol consumption in Durango City, Mexico. In this northern Mexican city, a low (6.1%) seroprevalence of *T. gondii* infection in the general population has been reported [25]. 

## 2. Results

Of the 1423 people with alcohol consumption studied, 224 (15.7%) had a history of suicidal ideation and 1199 (84.3%) did not have a history of suicidal ideation. Anti-*T. gondii* IgG antibodies were found in 34 of the 224 (15.2%) individuals with suicidal ideation and in 118 (9.8%) of the 1199 individuals without suicidal ideation. Seroprevalence of *T. gondii* infection was significantly higher in individuals with suicidal ideation than in individuals without suicidal ideation (OR: 1.63; 95% CI: 1.08–2.47; *p* = 0.01). Table 1 shows a stratification by age and sex and seroprevalence of *T. gondii* infection in individuals with and without suicidal ideation. Stratification by sex showed that women with suicidal ideation had a significantly higher (23/167: 13.8%) seroprevalence of *T. gondii* infection than women without suicidal ideation (53/799: 6.6%) (OR: 2.24; 95% CI: 1.33–3.78; *p* = 0.001). Whereas stratification by age groups showed that seroprevalence of *T. gondii* infection was significantly higher in individuals aged ≤30 and >50 years with suicidal ideation than individuals of the same age groups without suicidal ideation. 

High (>150 IU/mL) anti-*T. gondii* IgG antibody levels were found in 12 (5.4%) of the 224 individuals with suicidal ideation and in 50 (4.2%) of the 1199 individuals without suicidal ideation (OR: 1.30; 95% CI: 0.68–2.48; *p* = 0.42). Table 2 shows a stratification by sex and age groups of the study population and the association between high (>150 IU/mL) anti-*T. gondii* IgG antibody levels and suicidal ideation. Stratification by sex and age groups showed no association between the rates of high levels of anti-*T. gondii* antibodies and suicidal ideation.

Anti-*T. gondii* IgM was detected in 9 (26.5%) of 34 individuals with anti-*T. gondii* IgG antibodies and suicidal ideation and in 43 (36.4%) of 118 individuals with anti-*T. gondii* antibodies without suicidal ideation (OR: 0.62; 95% CI: 0.26–1.46; *p* = 0.28).

Concerning suicide attempts, of the 1423 people with alcohol consumption studied, 136 (9.6%) had a history of suicide attempts and 1287 (90.4%) did not have a history of suicide attempts. Anti-*T. gondii* IgG antibodies were found in 17 of the 136 (12.5%) individuals with suicide attempts and in 135 (10.5%) of the 1287 individuals without suicide attempts. Seroprevalence of *T. gondii* infection in individuals with suicide attempts (12.5%) was similar to that (10.5%) in individuals without suicide attempts (OR: 1.21; 95% CI: 0.71–2.08; *p* = 0.47). A stratification by age and sex and seroprevalence of *T. gondii* infection in individuals with and without suicide attempts is shown in Table 3. Women with suicide attempts had a significantly higher (13/101: 12.9%) seroprevalence of *T. gondii* infection than women without suicide attempts (63/865: 7.3%) (OR: 1.88; 95% CI: 0.99–3.55; *p* = 0.04). Stratification by age groups showed no association between the seropositivity to *T. gondii* and suicide attempts.

High (>150 IU/mL) anti-*T. gondii* IgG antibody levels were found in 5 (3.7%) of the 136 individuals with suicide attempts and in 57 (4.4%) of the 1287 individuals without suicide attempts (OR: 0.82; 95% CI: 0.32–2.09; *p* = 0.82). Table 4 shows a stratification by sex and age groups of the study population and the association between high (>150 IU/mL) anti-*T. gondii* IgG antibody levels and suicide attempts. Stratification by sex and age groups showed no association between the rates of high levels of anti-*T. gondii* antibodies and suicide attempts.

Anti-*T. gondii* IgM was detected in 5 (29.4%) of 17 individuals with anti-*T. gondii* IgG antibodies and suicide attempts and in 47 (34.8%) of 135 individuals anti-*T. gondii* IgG antibodies without suicide attempts. (OR: 0.78; 95% CI: 0.25–2.34; *p* = 0.78).

Suicide attempters have had from 1 to 20 suicide attempts (median: 1). Table 5 shows the association between the number of suicide attempts and rates of seropositivity and high levels of anti-*T. gondii* IgG antibodies. Seroprevalence of *T. gondii* infection or the rates of high anti-*T. gondii* IgG antibodies did not vary with the number of suicide attempts. 

Suicide attempters have attempted their last suicide attempt from 1 to 40 years (median: 5) before the interview. Seroprevalence of *T. gondii* infection or the rates of high anti-*T. gondii* IgG antibodies did not vary with the dates of suicide attempts (*p* = 0.81 and *p* = 0.81, respectively). Anti-*T. gondii* IgM was detected in 4 (57.1%) of 7 individuals with anti-*T. gondii* IgG antibodies who have committed their last suicide attempt during the last 3 years, and in none of 6 individuals with anti-*T. gondii* IgG antibodies who have committed their last suicide attempt 4 years or more ago (*p* = 0.06).

Methods of suicide attempt reported by suicide attempters included wrist cuts (*n* = 22), hanging (*n* = 15), drug overdose (*n* = 63), and other (*n* = 17) (firearm, poisoning, and throwing oneself to vehicles). Seroprevalence of *T. gondii* infection did not vary with the method of suicide attempt (*p* = 0.60). In contrast, the frequency of high anti-*T. gondii* antibodies was significantly higher in individuals with wrist cuts (3/22: 13.6%) than in individuals who used hanging, drug overdose, and other methods for suicide attempts (1/95: 1.1%) (OR: 14.84; 95% CI: 1.46–150.46; *p* = 0.02). The frequency of seropositivity to IgM among individuals with anti-*T. gondii* IgG antibodies was similar among the groups of methods used for suicide attempts (*p* = 0.34).

## 3. Discussion

Alcohol consumption and *T. gondii* exposure have been individually associated with suicidal behavior. However, there is a lack of information about the association between *T. gondii* exposure and suicidal behavior in persons with alcohol consumption. In the current study, we assessed the association between *T. gondii* exposure and suicidal behavior in a sample of people with alcohol consumption in Durango, Mexico. We found a significantly higher seroprevalence of *T. gondii* infection in individuals with suicidal ideation than in individuals without suicidal ideation. The 15.7% seroprevalence of *T. gondii* infection found in individuals with suicidal ideation in the current study is also higher than the one (6.1%) reported in the general population of Durango City [25]. Results thus suggest that *T. gondii* exposure is associated with suicidal ideation in individuals with alcohol consumption. This association was observed in women and individuals aged ≤30 years and >50 years. Our results are in line with another report on the association between *T. gondii* exposure and suicidal behavior. In a study of children and adolescents aged 11 to 18 years with depression, investigators found that seropositivity to *T. gondii* was associated with suicidal ideation [26]. In contrast, our results conflict with a negative association between *T. gondii* exposure and suicidal ideation in psychiatric patients suffering from mental and behavioral disorders due to psychoactive substance use [27]. However, in that study, only 38 patients with alcohol use were studied. In a National Health and Nutrition Examination Survey with data collected between 2009 and 2012 that included subjects aged 20 to 80 years in the USA, researchers found no association between suicidal ideation and anti-*T. gondii* antibody titer [28]. Differences in the association among the studies could be due to differences in the characteristics of the study population. We studied only people with alcohol consumption whereas researchers in the American survey studied the general population. In addition, risk factors for *T. gondii* infection and suicidal behavior might be different among the study populations. In the present study, exposure to *T. gondii* was not associated with suicidal ideation in the middle age group (31–50 years). It is unclear why there was not an association in this age group. It raises the question of whether this age group could be more resistant to the possible behavioral effects induced by *T. gondii* than the younger and older age groups. With respect to suicide attempts, we found an association between *T. gondii* seropositivity and suicide attempts in women but not in men or the whole population studied. It is not clear why this association was found in women but not in men. It is possible that the greater association of *T. gondii* exposure and suicidal behaviors found more in women rather than in men could be due to the difference in risk factors, such as type of work, diet, contact with animals, and others among the gender groups. The 9.6% seroprevalence of *T. gondii* infection found in individuals with suicide attempts in the current study is slightly higher than the one (6.1%) reported in the general population of Durango City [25]. The lack of association between suicide attempts and *T. gondii* seropositivity found in the whole study population in this survey agrees with results found in other studies. A lack of association between *T. gondii* seropositivity and suicide attempts was reported in patients with mood disorders in the USA [13] and in psychiatric outpatients in Mexico [15]. In contrast, in a study in Turkey, researchers found a significantly higher rate of *T. gondii* seropositivity in suicide attempters than in healthy volunteers [29]. On the other hand, the association between *T. gondii* exposure and suicide attempts in women found in the current study agrees with that found in a study in the USA where researchers observed a positive relationship between rates of infection with *T. gondii* and suicide in women of postmenopausal age [14]. In a study of schizophrenic patients in Iran, investigators found a lower rate of suicide attempts in *T. gondii* seropositive male patients than in seronegative ones [30]. In the present study, no association between the frequency of high anti-*T. gondii* IgG antibody levels and suicide attempts was found. This result conflicts with those reported in other studies. An association between the frequency of high anti-*T. gondii* antibody levels and suicide attempts were found in psychiatric outpatients in Mexico [15], patients with recurrent mood disorders in the USA [13], and schizophrenic patients younger than 38 years in Germany [17]. The difference in the findings among the studies could be due to differences in the characteristics of the participants among the studies. It seems that the association between high anti-*T. gondii* antibody levels and suicide attempts is more readily observed in psychiatric patients than in people with alcohol consumption. In the current study, no association between *T. gondii* seropositivity or serointensity and number of suicide attempts was found. The lack of this association was also reported in a study of patients with recurrent mood disorders [13]. In contrast, a study in psychiatric outpatients found that the seroprevalence of *T. gondii* infection increased with the number of suicide attempts [15]. Interestingly, we found that the frequency of high anti-*T. gondii* antibodies was significantly higher in individuals with wrist cuts than in individuals who used other methods for suicide attempts. Further research to determine the association between *T. gondii* serointensity and methods for suicide attempts should be conducted.

This study has some limitations: (1) we considered a qualitative (history of alcohol consumption) rather than a quantitative (number and type of alcoholic drinks) criterium for enrollment of participants; and (2) we could not trace back the date of infection and correlated it with suicidal behavior. Therefore, further research to determine the relationship between *T. gondii* exposure and: (1) the quantity of alcohol consumption, and (2) the time of having a suicidal behavior episode is needed. The association of suicidal behavior and *T. gondii* exposure found in the present study should not be concluded as categorical due to the possible presence of interactions of different risk factors and the absence of knowledge and characteristics that would be important to determine the certainty of this epidemiological link. Additional and more complete studies to confirm the association between *T. gondii* exposure and suicidal behavior in alcohol consumers should be conducted.

## 4. Materials and Methods

We performed a cross-sectional study of 1423 people with alcohol consumption in Durango, Mexico from 2014 to 2019. The following inclusion criteria were established: people with a history of alcohol consumption (at least one alcoholic drink every month for the last six months) and any gender, aged ≥15 years, with informed consent. In this exploratory study, the quantity of alcohol consumption was not considered for analysis. Birthplace and residence place were not restrictive criteria for enrollment. Participants were enrolled at public health institutions (hospitals and health care units). Participation was voluntary without compensation. Data were collected and revised by our research team with the aid of the laboratory personnel of health institutions and students.

Face-to-face interviews were conducted and a questionnaire was used to record the sociodemographic and clinical data of participants. Sociodemographic data of participants are shown in Table 6. The mean age of the study population was 38.13 ± 12.99 (range 15–84) years. There were more women than men because women were more willing to participate than men. Most (1010: 71.0%) participants were healthy, 408 (28.7%) were ill, and in 5 (0.3%) no information about health status was recorded. No information about the treatment of psychiatric disorders was obtained. None of the participants reported having an alcohol use disorder. Suicidal behavior items include a history of suicidal ideation, suicide attempts, number of suicide attempts, date of last suicide attempt, and method of suicide attempt.

A blood sample from each participant was obtained. Blood was centrifuged and serum was obtained and stored at −20 °C until analyzed. Serum samples were tested for anti-*T. gondii* IgG antibodies using a commercially available enzyme immunoassay “*Toxoplasma gondii* IgG” kit (Diagnostic Automation/Cortez Diagnostics, Inc., Woodland Hills, CA, USA). Serum samples with anti-*T. gondii* IgG antibodies were further tested for anti-*T. gondii* IgM antibodies by a commercially available enzyme immunoassay, the “*Toxoplasma gondii* IgM” kit (Diagnostic Automation/Cortez Diagnostics, Inc.). All assays were performed following the manufacturer’s instructions.

The data were analyzed with the software Epi Info version 7 and IBM SPSS Statistics version 20. The Pearson’s chi-square test and the Fisher exact test (when values were less than 5) were used to compare the frequencies among the groups. The sample size (*n* = 941) was calculated using a population size of 100,000, a 99% confidence level, an expected frequency of *T. gondii* infection of 6.1% [25], and 2% of confidence limits. Therefore, the number of participants (*n* = 1423) was representative of the larger population from which it was collected. Odd ratios (OR) and 95% confidence intervals (CI) were calculated, and the statistical significance was set at a *p* value < 0.05.

## 5. Conclusions

The results of this study suggest that *T. gondii* seropositivity is associated with suicidal behavior in people with alcohol consumption. Results of this first study on the association between *T. gondii* exposure and suicidal behavior in people with alcohol consumption warrant further research.

## Figures and Tables

**Table 1:** Stratification by sex and age and the association between *T. gondii* exposure and suicidal ideation in people with alcohol consumption.

	History of Suicidal Ideation			No History of Suicidal Ideation					
		Seropositivity			Seropositivity			95%	
	No.	to *T. gondii*		No.	to *T. gondii*			Confidence	*p*
Characteristic	Tested	No.	%	Tested	No.	%	OR	Interval	Value
Sex									
Male	57	11	19.3	400	65	16.2	1.23	0.60–2.50	0.56
Female	167	23	13.8	799	53	6.6	2.24	1.33–3.78	0.001
Age (years)									
≤30	60	10	16.7	389	27	6.9	2.68	1.22–5.87	0.01
31–50	127	15	11.8	583	68	11.7	1.01	0.55–1.83	0.96
>50	37	9	24.3	227	23	10.1	2.85	1.19–6.77	0.01
All	224	34	15.2	1199	118	9.8	1.63	1.08–2.47	0.01

**Table 2:** Stratification by sex and age and the association between high (>150 IU/mL) levels of anti-*T. gondii* IgG and suicidal ideation in people with alcohol consumption.

	History of Suicidal Ideation			No History of Suicidal Ideation					
		>150 IU/mL			>150 IU/mL			95%	
	No.	of IgG		No.	of IgG			Confidence	*p*
Characteristic	Tested	No.	%	Tested	No.	%	OR	Interval	Value
Sex									
Male	57	5	8.8	400	26	6.5	1.38	0.50–3.76	0.57
Female	167	7	4.2	799	24	3.0	1.41	0.59–3.33	0.42
Age (years)									
≤30	60	3	5.0	389	7	1.8	2.87	0.72–11.42	0.13
31–50	127	5	3.9	583	32	5.5	0.70	0.26–1.84	0.65
>50	37	4	10.8	227	11	4.8	2.38	0.71–7.91	0.23
All	224	12	5.4	1199	50	4.2	1.30	0.68–2.48	0.42

**Table 3:** Stratification by sex and age and the association between *T. gondii* exposure and suicide attempts in people with alcohol consumption.

	Suicide Attempts			No Suicide Attempts					
		Seropositivity			Seropositivity			95%	
	No.	to *T. gondii*		No.	to *T. gondii*			Confidence	*p*
Characteristic	Tested	No.	%	Tested	No.	%	OR	Interval	Value
Sex									
Male	35	4	11.4	422	72	17.1	0.62	0.21–1.83	0.48
Female	101	13	12.9	865	63	7.3	1.88	0.99–3.55	0.04
Age (years)									
≤30	41	3	7.3	408	34	8.3	0.86	0.25–2.96	1.00
31–50	70	9	12.9	640	74	11.6	1.12	0.53–2.36	0.74
>50	25	5	20.0	239	27	11.3	1.96	0.68–5.65	0.20
All	136	17	12.5	1287	135	10.5	1.21	0.71–2.08	0.47

**Table 4:** Stratification by sex and age and the association between high (>150 IU/mL) levels of anti-*T. gondii* IgG and suicide attempts in people with alcohol consumption.

	Suicide Attempts			No Suicide Attempts					
		>150 IU/mL			>150 IU/mL			95%	
	No.	of IgG		No.	of IgG			Confidence	*p*
Characteristic	Tested	No.	%	Tested	No.	%	OR	Interval	Value
Sex									
Male	35	1	2.9	422	30	7.1	0.38	0.05–2.90	0.49
Female	101	4	4.0	865	27	3.1	1.27	0.43–3.73	0.55
Age (years)									
≤30	41	1	2.4	408	9	2.2	1.10	0.13–8.97	1.00
31–50	70	2	2.9	640	35	5.5	0.50	0.11–2.16	0.56
>50	25	2	8.0	239	13	5.4	1.51	0.32–7.11	0.64
All	136	5	3.7	1287	57	4.4	0.82	0.32–2.09	0.82

**Table 5:** Association between number of suicide attempts, rates of seropositivity, and high (>150 IU/mL) levels of anti-*T. gondii* IgG antibodies.

	No. of	Prevalence of			High Levels of		
No. of	Subjects	*T. gondii* Infection			*T. gondii* Antibodies		
Suicide Attempts	Tested	No.	%	*p*	No.	%	*p*
1	71	9	12.7	0.22	1	1.4	0.09
2	31	2	6.5		2	6.5	
3	17	3	17.6		1	5.9	
4	3	1	33.3		0	0	
5	3	1	33.3		0	0	
6	1	1	100		1	100	
7	1	0	0		0	0	
10	1	0	0		0	0	
12	1	0	0		0	0	
20	1	0	0		0	0	

**Table 6:** Socio-demographic characteristics of the study population.

	Subjects Tested	
Characteristic	No.	%
Age Groups (Years)		
30 or less	449	31.6
31–50	710	49.9
>50	264	18.5
Gender		
Male	457	32.1
Female	966	67.9
Birthplace		
Durango State	1270	89.3
Other Mexican State	147	10.3
Abroad	6	0.4
Residence place		
Durango State	1411	99.2
Other Mexican State	11	0.7
Abroad	1	0.1
Residence area		
Urban	1109	77.9
Suburban	167	11.8
Rural	147	10.3
Educational level		
No education	14	1.0
1 to 6 years	203	14.3
7–12 years	729	51.2
>12 years	477	33.5
Socio-economic level		
Low	337	23.7
Medium	1079	75.8
High	7	0.5

## Data Availability

Data are provided within the article.
